# A Randomized Study to Evaluate the Efficacy of Tele-Follow-Up Compared With Standard Follow-Up in Patients Undergoing Coronary Angioplasty and Stenting: A Six-Month Study at a Tertiary Care Hospital

**DOI:** 10.7759/cureus.114865

**Published:** 2026-08-20

**Authors:** Kishan Mavani, Barun Kumar, Ravi Kant, Vijay Krishnan, Meenu Singh

**Affiliations:** 1 Department of Cardiology, All India Institute of Medical Sciences, Rishikesh, Rishikesh, IND; 2 Department of Internal Medicine, All India Institute of Medical Sciences, Rishikesh, Rishikesh, IND; 3 Department of Psychiatry, All India Institute of Medical Sciences, Rishikesh, Rishikesh, IND; 4 Department of Pediatrics, All India Institute of Medical Sciences, Rishikesh, Rishikesh, IND

**Keywords:** follow-up, percutaneous coronary intervention, randomized controlled trial, secondary prevention, tele-consultation, telemedicine

## Abstract

Background

Coronary artery disease (CAD) remains a major cause of morbidity and mortality worldwide. Despite advances in percutaneous coronary intervention (PCI), recurrent cardiovascular events remain common due to gaps in secondary prevention and long-term follow-up. Telemedicine offers a potential solution by improving access to care, patient engagement, and risk-factor monitoring.

Methods

This single-center, open-label randomized controlled trial included 106 patients undergoing PCI at All India Institute of Medical Sciences (AIIMS), Rishikesh, India, who were randomized 1:1 to structured tele-follow-up (WhatsApp, SMS, and audio/video consultations) or standard outpatient follow-up. Of these, 100 participants completed follow-up and were included in the available-case analysis. The primary outcome was major adverse cardiovascular events (MACEs) assessed at prespecified one-, three-, and six-month follow-up assessments. Secondary outcomes included medication adherence, lipid control, blood pressure control, glycemic control, lifestyle modification, and hospital readmissions.

Results

The mean age was 55.5 ± 9.2 years, and 88% were male. MACE rates were comparable between groups throughout follow-up (p > 0.05). Tele-follow-up achieved significantly lower low-density lipoprotein cholesterol (LDL-C) levels at one month (69.72 vs. 87.08 mg/dL, p = 0.003) and at six months (53.90 vs. 63.58 mg/dL, p = 0.014). Blood pressure control was generally comparable between groups, with an isolated lower diastolic blood pressure in the tele-follow-up group at one month that was not sustained at subsequent follow-up. Medication adherence was ≥94% in both groups throughout follow-up. No significant differences were observed in HbA1c, systolic blood pressure, bleeding events, stent failure, or mortality.

Conclusions

Structured tele-follow-up after PCI was feasible, with observed short-term clinical outcomes comparable to standard follow-up. It was associated with better lipid control, while blood pressure control was broadly similar between the groups. Tele-follow-up may therefore be a practical and scalable follow-up strategy after PCI, particularly in resource-limited settings. Larger multicenter studies with longer follow-up are needed to confirm its impact on clinical outcomes.

## Introduction

Coronary heart disease (CHD) remains the leading cause of mortality worldwide, with cardiovascular diseases responsible for approximately 19.2 million deaths in 2023 and projected to exceed 35 million deaths annually by 2050. More than 75% of these deaths occur in low- and middle-income countries, highlighting substantial disparities in access to cardiovascular care and preventive services [1,2].

India has undergone a rapid epidemiological transition, with coronary artery disease (CAD) now accounting for nearly one-third of all deaths and occurring at a younger age than in Western populations [3-5]. The expanding use of percutaneous coronary intervention (PCI) has substantially improved acute management of CAD [6,7]. However, long-term outcomes remain dependent on effective secondary prevention through sustained medication adherence, cardiovascular risk factor control, and lifestyle modification. Conventional outpatient follow-up is frequently limited by geographic barriers, overcrowded healthcare facilities, socioeconomic constraints, and shortages of specialist healthcare providers, contributing to suboptimal long-term care [8].

Telemedicine has emerged as a practical strategy to overcome these challenges by facilitating remote follow-up, medication support, lifestyle counseling, and timely clinical assessment [9]. Although studies from high-income countries have demonstrated its safety, effectiveness, and cost-effectiveness in cardiovascular care [10-12], evidence from randomized trials in India remains limited.

The primary objective of the present study was to compare structured tele-follow-up with standard in-person follow-up after PCI with respect to major adverse cardiovascular events (MACEs) and selected cardiovascular risk-factor outcomes. The study was designed as a comparative randomized study and was not prospectively intended to establish superiority, equivalence, or non-inferiority of tele-follow-up.

## Materials and methods

Study design and setting

This was a single-center, open-label, parallel-group randomized controlled trial conducted in the Department of Cardiology, All India Institute of Medical Sciences (AIIMS), Rishikesh, India. The study was conducted from February 2024 to July 2025. Patient screening and recruitment were performed from February 2024 to January 2025, followed by six months of follow-up after enrolment of the last participant. The study was approved by the Institutional Research and Ethics Committee prior to initiation.

Study population

Patients admitted to the Department of Cardiology who underwent PCI with stent implantation during the index hospitalization were screened for eligibility. 

Inclusion criteria

Patients aged 18-70 years with CAD (chronic coronary syndrome, unstable angina, non-ST-elevation myocardial infarction (NSTEMI), or ST-elevation myocardial infarction (STEMI)) who underwent PCI with stent implantation during the index hospitalization were eligible for inclusion. Patients were required to provide written informed consent, be able to communicate through WhatsApp, and be able to speak English or Hindi.

Exclusion criteria

Patients were excluded if they had significant arrhythmias; severe heart failure (NYHA Class III-IV) or left ventricular ejection fraction (LVEF) <40%; residual coronary disease requiring staged PCI; major comorbidities requiring frequent hospital visits, including severe anemia, cerebrovascular disease, chronic liver disease, chronic kidney disease, or uncontrolled diabetes; inability to use a smartphone because of visual, auditory, cognitive, or motor impairment; lack of internet access at residence; or inability or unwillingness to provide informed consent.

The exclusion criteria were primarily intended to ensure the safety and feasibility of the structured tele-follow-up strategy and to avoid including patients requiring more intensive clinical assessment or frequent hospital-based care.

Data collection

All patients admitted with CAD who underwent PCI were screened for eligibility. Eligible patients were approached during hospitalization, and those expressing interest underwent a face-to-face interview. Written informed consent was obtained before enrolment. Baseline demographic, clinical, laboratory, and angiographic data were recorded.

Randomization and allocation concealment

Participants were randomized in a 1:1 ratio to either the tele-follow-up group or the standard follow-up group using block randomization with a fixed block size of four. Allocation concealment was maintained using opaque sealed envelopes, which were opened after enrolment and eligibility confirmation in the presence of a nursing officer and a senior resident.

Study intervention and follow-up

Participants in the tele-follow-up group received structured follow-up assessments at one, three, and six months after PCI through WhatsApp, SMS, and audio/video communication. Each assessment included evaluation of cardiovascular symptoms and clinical events, medication adherence, lifestyle modification, blood pressure, lipid profile, and glycated hemoglobin (HbA1c). The approximate duration of each tele-follow-up consultation was four to seven minutes.

Participants in the standard follow-up group underwent routine in-person outpatient follow-up at the same one-, three-, and six-month intervals, with the same clinical and biochemical assessment domains.

The principal difference between the groups was the mode of follow-up, with structured remote assessment in the tele-follow-up group and in-person outpatient assessment in the standard follow-up group.

Study hypothesis and objective

The primary objective of the study was to compare structured tele-follow-up with standard in-person follow-up after PCI with respect to MACEs and selected cardiovascular risk-factor outcomes. The study was designed as a comparative randomized study and was not prospectively intended to establish superiority, equivalence, or non-inferiority of tele-follow-up.

Outcomes

Primary Outcome

The primary outcome was MACE, defined as a composite of acute myocardial infarction, non-fatal stroke, cardiovascular death, unstable angina requiring hospitalization, and heart failure requiring hospitalization.

MACE was assessed at the prespecified one-, three-, and six-month follow-up assessments and reported as the proportion of participants experiencing MACE at each respective follow-up assessment. The analysis was based on the observed outcomes at the specified follow-up assessments rather than a formal time-to-event or recurrent-event analysis.

Secondary Outcomes

Secondary outcomes included medication adherence, major bleeding events, stent failure including stent thrombosis or in-stent restenosis, hospital readmission for any cardiovascular cause, all-cause mortality, and cardiovascular risk-factor control.

The proportion of participants achieving prespecified risk-factor targets was also assessed, including low-density lipoprotein cholesterol (LDL-C) <55 mg/dL, HbA1c <7%, and blood pressure <130/80 mmHg.

Sample size calculation

The sample size was estimated prospectively using a prevalence-based calculation according to the following formula: $N = \frac{(Z_{\mathrm{crit}} + Z_{\mathrm{pwr}})^2 \times P \times Q}{L^2}$, where N is the required sample size, Zcrit is the standard normal variate corresponding to the critical significance level, Zpwr is the standard normal variate corresponding to the statistical power, P is the estimated prevalence, Q = 1 - P, and L is the absolute error. For the present study, Zcrit was 2.576, Zpwr was 1.645, P was 5%, Q was 95%, and the absolute error was 10%. Based on these prespecified parameters, the calculated sample size was approximately 85 participants. A target sample of at least 100 participants was therefore planned, and 106 participants were ultimately randomized. This calculation was not based on a prespecified between-group difference in MACE or a non-inferiority margin; therefore, the study was not formally powered to establish equivalence or non-inferiority for clinical events.

Statistical analysis

Statistical analyses were performed using IBM SPSS Statistics for Windows, Version 25 (Released 2017; IBM Corp., Armonk, NY, USA). Continuous variables were compared using Student's t-test or Mann-Whitney U test, as appropriate, while categorical variables were compared using the chi-square test or Fisher's exact test, as appropriate. A two-sided p-value <0.05 was considered statistically significant.

The randomized groups were compared at the prespecified one-, three-, and six-month follow-up assessments using the above statistical tests. The analysis was based on participants with available follow-up data. Of the 106 randomized participants, 100 completed the planned follow-up and were included in the available-case analysis. The six participants who did not complete follow-up were not assigned clinical outcomes in the absence of available outcome data.

The study was not designed as a formal non-inferiority trial, and no non-inferiority margin was prespecified. Accordingly, the results were interpreted as observed between-group comparisons rather than as evidence of superiority, equivalence, or non-inferiority.

## Results

Between February 2024 and January 2025, a total of 164 consecutive patients undergoing PCI were screened for eligibility. Of these, 106 patients (64.6%) fulfilled the inclusion criteria and were randomized, while 58 patients (35.4%) were excluded.

**Figure 1:**
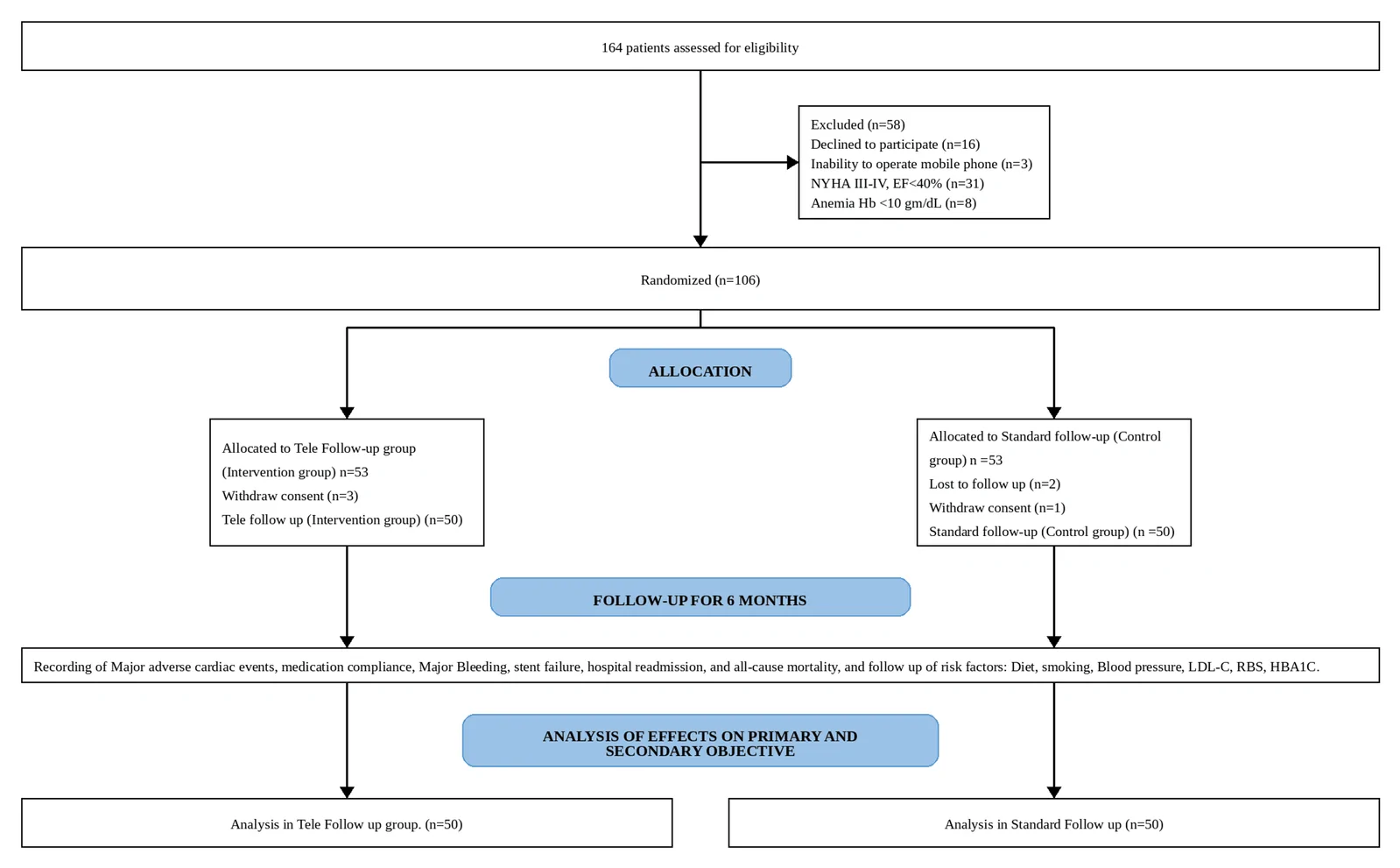
Consort diagram for the study

Figure 1 illustrates the study flow chart. The 106 patients who met the inclusion criteria were randomized in a 1:1 ratio to the tele-follow-up group or the standard follow-up group. During follow-up, three patients in each group were lost to follow-up or withdrew consent. Therefore, 100 patients (50 per group) completed follow-up and were included in the available-case analysis.

Baseline demographic and clinical characteristics were comparable between the tele-follow-up and standard follow-up arms (Table 1). Mean age (56.54 ± 8.97 vs. 54.5 ± 9.51 years; p = 0.272; independent-samples t-test) and gender distribution (88% male; n = 44 in both arms; p = 1; chi-square test) were similar, and all patients were married in both groups. Socioeconomic status (p = 0.156; Mann-Whitney U test) and distance from hospital (187.18 ± 401.9 vs. 104.18 ± 150.34 km; p = 0.852; Mann-Whitney U test) also did not differ significantly.

**Table 1:** Baseline characteristics of the tele-follow-up and standard-follow-up arms Data are presented as mean ± SD, n (%), or median (IQR), as appropriate. †Chi-square test; *Mann-Whitney U test; ‡Independent-samples t-test. LDL-C: Low-Density Lipoprotein Cholesterol; HDL-C: High-Density Lipoprotein Cholesterol; LVEF: Left Ventricular Ejection Fraction; CAD: Coronary Artery Disease; STEMI: ST-Elevation Myocardial Infarction; NSTEMI: Non-ST-Elevation Myocardial Infarction; CCS: Chronic Coronary Syndrome; CAG: Coronary Angiography; SVD: Single Vessel Disease; DVD: Double Vessel Disease; TVD: Triple Vessel Disease; LM+TVD: Left Main + Triple Vessel Disease

Variables	Tele-follow-up arm (n=50)	Standard follow-up (n=50)	p-value
Demographics			
Age (y), Mean (SD)	56.54 (8.97)	54.5 (9.51)	0.272
Sex (Male), n (%)	44 (88)	44 (88)	1^†^
Sex (Female), n (%)	6 (12)	6 (12)	1^†^
Marital Status			
Married	50 (100)	50 (100)	-
Socioeconomic Status			
Upper	13 (26)	5 (10)	0.156*
Upper Middle	10 (20)	14 (28)	
Middle	21 (42)	20 (40)	
Lower Middle	6 (12)	10 (20)	
Lower	0 (0)	1 (2)	
Distance From Hospital (km)	187.18 (401.9)	104.18 (150.34)	0.852
Clinical Data			
Comorbid Condition			
Hypertension	20 (40)	21 (42)	0.839^†^
Diabetes	12 (24)	14 (28)	0.648
Lifestyle			
Healthy Diet	32 (64)	27 (54)	0.312
Current Smoking	18 (36)	18 (36)	1
Current Drinking	4 (8)	13 (26)	0.017
BMI (Kg/m^2^)	24.49 (3.66)	24.67 (4.14)	0.81^‡^
Blood Pressure (mmHg)			
Systolic	124.5 (12.63)	122.7 (15.17)	0.521^‡^
Diastolic	74.36 (8.42)	74.14 (7.84)	0.893^‡^
Hemoglobin (gm/dL)	13.09 (1.13)	13.34 (1.9)	0.441^‡^
HbA1c (%)	6.67 (1.53)	6.71 (1.72)	0.906^‡^
Blood Lipids (mg/dL)			
Total Cholesterol	155.5 (43.63)	157.3 (55.18)	0.857^‡^
Triglyceride	156.3 (96.5)	151.6 (57.88)	0.768^‡^
LDL-C	82.14 (38.73)	88.57 (47.73)	0.463^‡^
HDL-C	40.93 (15.91)	39.54 (9.69)	0.598^‡^
LVEF (%), median (IQR)	50 (20)	47.50 (15)	0.222*
Type of CAD			
STEMI	30 (60)	23 (46)	0.489^†^
NSTEMI	5 (10)	9 (18)	
Unstable Angina	4 (8)	4 (8)	
CCS	11 (22)	14 (28)	
CAG			
SVD	10 (20)	11 (22)	0.933
DVD	15 (30)	16 (32)	
TVD	23 (46)	22 (44)	
LM+TVD	2 (4)	1 (2)	

Comorbidities, including hypertension (n = 20, 40% vs. n = 21, 42%; p = 0.839; chi-square test) and diabetes (n = 12, 24% vs. n = 14, 28%; p = 0.648; chi-square test), and lifestyle factors were comparable between groups, except for current alcohol consumption, which was significantly higher in the standard follow-up arm (n = 13, 26% vs. n = 4, 8%; p = 0.017; chi-square test). Healthy diet (n = 32, 64% vs. n = 27, 54%; p = 0.312; chi-square test) and smoking (36% vs. 36%; p = 1.000; chi-square test) did not show significant differences. At six months, fewer patients in the tele-follow-up group continued smoking (6% vs. 18%), although the difference was not statistically significant (p = 0.23). Adherence to a healthy diet was numerically higher in the tele-follow-up group at all follow-up assessments, but none of the between-group differences reached statistical significance. Anthropometric and biochemical parameters, including BMI, blood pressure, hemoglobin, HbA1c, and lipid profile, were statistically similar between the arms (all p > 0.05; independent-samples t-test). Median LVEF (IQR) was 50% (20) in the tele-follow-up group and 47.5% (15) in the standard follow-up group, with no significant difference (p = 0.222; Mann-Whitney U test).

Type of CAD and extent of disease on coronary angiography were also comparable between groups (p = 0.489 and p = 0.933, respectively; chi-square test). Overall, baseline characteristics were well balanced between arms, with current alcohol consumption being the only variable showing a statistically significant difference.

**Figure 2:**
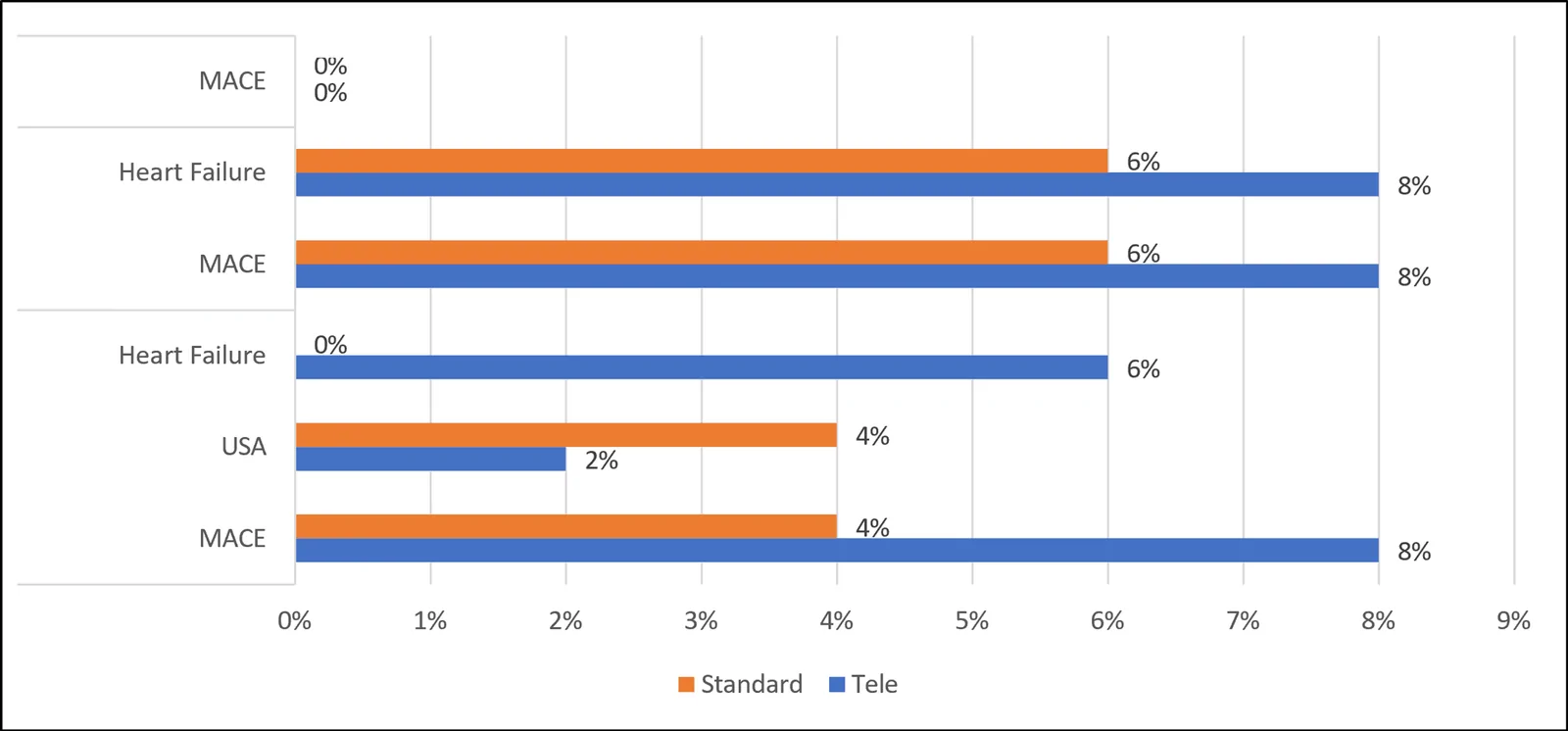
Comparison of MACE events between the tele-follow-up and standard-follow-up arms at 1, 3, and 6 months MACE: Major Adverse Cardiovascular Event

The primary composite endpoint of MACE was observed in a small proportion of participants in both groups at the prespecified follow-up assessments. No statistically significant between-group difference was observed during follow-up (Table 2 and Figure 2). At one month, MACE was recorded in four (8%) patients in the tele-follow-up arm versus two (4%) in the standard follow-up arm (p = 0.678; chi-square test). At three months, MACE was recorded in four (8%) versus three (6%) patients, respectively (p = 0.505; chi-square test), and no new MACE events were recorded in either arm at the six-month assessment. Individual components of the composite endpoint, including acute myocardial infarction, unstable angina, non-fatal stroke, and cardiovascular death, occurred infrequently and did not differ meaningfully between the groups at any time point. Heart failure hospitalization was numerically more common in the tele-follow-up arm at one month (3 (6%) vs. 0 (0%)) and at three months (4 (8%) vs. 3 (6%)), with no new heart failure hospitalizations recorded in either arm at the six-month assessment.

**Table 2:** Primary and secondary outcome analysis at 1, 3, and 6 months in both groups Data are presented as n (%) or mean ± SD, as appropriate. *MACE values represent events recorded at each respective follow-up assessment and are not cumulative event rates. MACE: Major Adverse Cardiovascular Event; LDL: Low-Density Lipoprotein

Follow up at 1 month				Follow up at 3 months			Follow up at 6 months		
Variables	Tele-follow-up arm (n=50)	Standard follow-up (n=50)	p-value	Tele-follow-up arm (n=50)	Standard follow-up (n=50)	p-value	Tele-follow-up arm (n=50)	Standard follow-up (n=50)	p-value
Primary end point									
MACE*	4 (8)	2 (4)	0.678	4 (8)	3 (6)	0.505	0 (0)	0 (0)	-
Acute MI	0 (0)	0 (0)	-	0 (0)	0 (0)	-	0 (0)	0 (0)	-
Unstable angina	1 (2)	2 (4)	-	0 (0)	0 (0)	-	0 (0)	0 (0)	-
Heart failure	3 (6)	0 (0)	-	4 (8)	3 (6)	-	0 (0)	0 (0)	-
Non-fatal stroke	0 (0)	0 (0)	-	0 (0)	0 (0)	-	0 (0)	0 (0)	-
Cardiovascular death	0 (0)	0 (0)	-	0 (0)	0 (0)	-	0 (0)	0 (0)	-
Secondary end point									
Major bleeding	0 (0)	0 (0)	-	0 (0)	0 (0)	-	0 (0)	0 (0)	-
Stent failure	0 (0)	0 (0)	-	0 (0)	0 (0)	-	0 (0)	0 (0)	-
Hospital readmission	3 (6)	5 (10)	0.715	0 (0)	0 (0)	-	0 (0)	0 (0)	-
All-cause mortality	0 (0)	0 (0)	-	0 (0)	0 (0)	-	0 (0)	0 (0)	-
Medication compliance	49 (98)	50 (100)	1	48 (96)	47 (94)	0.92	50 (100)	50 (100)	1
Smoking	18 (36)	19 (38)	0.841	17 (34)	17 (34)	1	3 (6)	9 (18)	0.23
Healthy diet	34 (68)	27 (54)	0.157	43 (86)	29 (58)	0.06	47 (94)	41 (82)	0.08
LDL (mg/dL)	69.72 ± 28.10	87.08 ± 29.12	0.003	63.98 ± 23.62	68.8 ± 30.61	0.38	53.9 ± 14.54	63.58 ± 23.24	0.014
LDL (<55 mg/dL)	10 (20)	4 (8)	0.143	16 (32)	10 (20)	0.23	28 (56)	20 (40)	0.109
HBA1C; mean (SD)	6.5 ± 1.53	6.43 ± 1.72	0.83	6.19 ± 0.93	6.1 ± 1.02	0.903	5.98 ± 0.72	6.03 ± 0.82	0.768
HBA1C (<7 %)	38 (76)	38 (76)	1	40 (80)	44 (88)	0.275	46 (92)	44 (88)	0.505
Systolic blood pressure; mean (SD)	124.4 ± 12.04	124.28 ± 13.63	0.96	123.88 ± 10.29	120.02 ± 13.27	0.107	123.94 ± 11.78	120.56 ± 14.81	0.21
Diastolic blood pressure; mean (SD)	70.04 (8.45)	74.88 (7.60)	0.03	73.08 (6.91)	71.94 (7.14)	0.419	72.16 (6.17)	71.76 (7.71)	0.775
Systolic (<130 mmHg)	28 (56)	33 (66)	0.308	33 (66)	38 (76)	0.27	29 (58)	37 (74)	0.139
Diastolic (<80 mmHg)	43 (86)	37 (74)	0.141	37 (74)	34 (68)	0.549	42 (84)	39 (78)	0.61

Among secondary safety endpoints, no episodes of major bleeding, stent failure, or all-cause mortality were observed in either arm across the study period. Hospital readmission occurred in three (6%) patients in the tele-follow-up arm versus five (10%) patients in the standard follow-up arm at one month, with no further readmissions in either arm at three or six months (p = 0.715; chi-square test).

**Figure 3:**
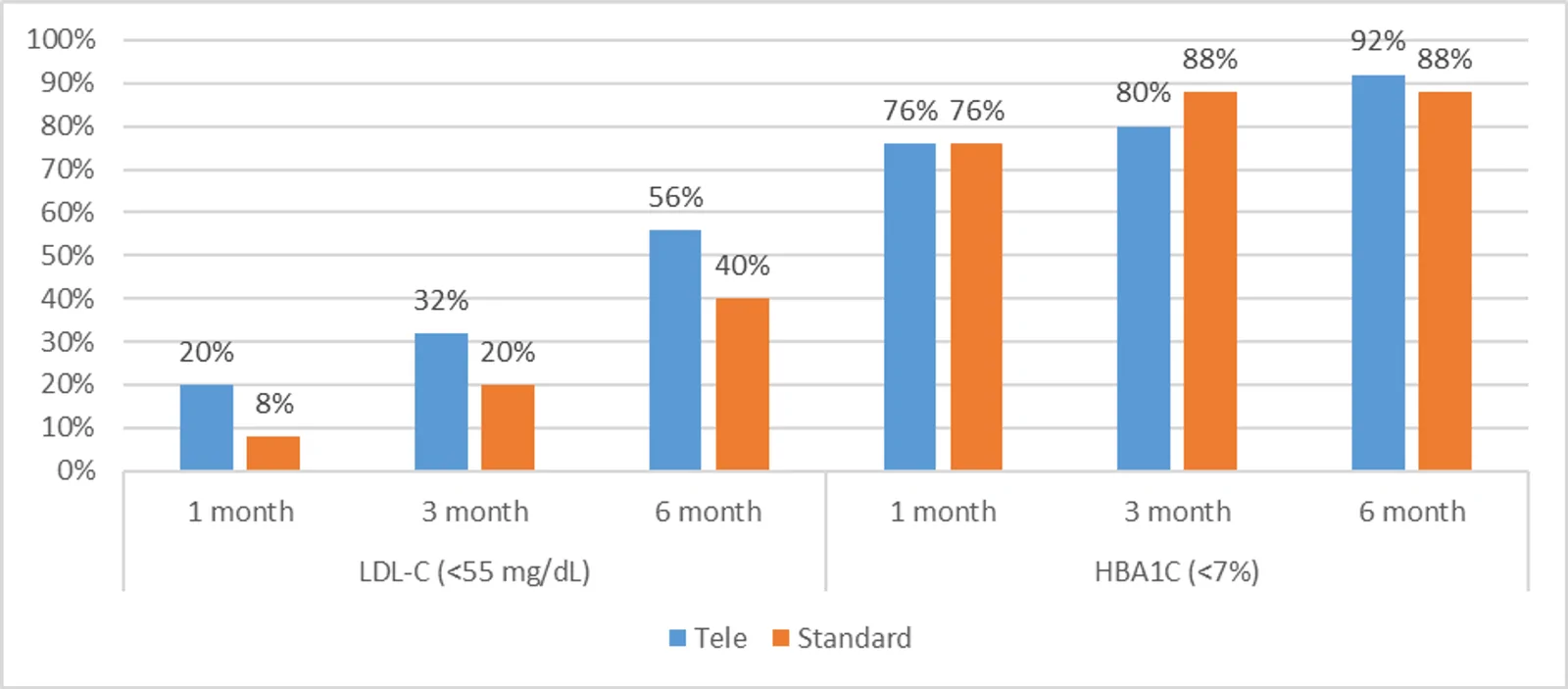
Comparison of LDL-C (<55 mg/dL) and HbA1c level (<7%) between the tele and standard arms LDL-C: Low-Density Lipoprotein Cholesterol

Regarding lipid control, mean LDL-C was significantly lower in the tele-follow-up arm at one month (69.72 ± 28.10 vs. 87.08 ± 29.12 mg/dL; p = 0.003) and six months (53.9 ± 14.54 vs. 63.58 ± 23.24 mg/dL; p = 0.014), though not at three months (63.98 ± 23.62 vs. 68.8 ± 30.61 mg/dL; p = 0.38; independent-samples t-test). The proportion of patients achieving the intensive LDL-C target of <55 mg/dL was numerically higher in the tele-follow-up group at all follow-up assessments; however, the between-group differences were not statistically significant. Glycemic control was similar between arms throughout, with comparable mean HbA1c at one month (6.5 ± 1.53% vs. 6.43 ± 1.72%; p = 0.83), three months (6.19 ± 0.93% vs. 6.1 ± 1.02%; p = 0.903), and six months (5.98 ± 0.72% vs. 6.03 ± 0.82%; p = 0.768; independent-samples t-test). Similarly, the proportions of patients achieving HbA1c <7% were comparable at one month (n = 38, 76% vs. n = 38, 76%; p = 1.000), three months (n = 40, 80% vs. n = 44, 88%; p = 0.275), and six months (n = 46, 92% vs. n = 44, 88%; p = 0.505; chi-square test) (Figure 3).

## Discussion

This randomized controlled trial evaluated structured tele-follow-up compared with standard in-person follow-up after PCI over a six-month period. No statistically significant between-group difference in MACE was observed during follow-up. Tele-follow-up was associated with lower mean LDL-C levels at one and six months, while other cardiovascular risk-factor outcomes were largely comparable between groups. These findings should be interpreted cautiously given the small sample size, limited number of clinical events, and selected study population.

The primary endpoint of the study, MACE, occurred infrequently and at comparable rates in both study arms, with no new events recorded in either group at the six-month assessment. No clear difference in short-term cardiovascular outcomes was observed between structured tele-follow-up and conventional in-person follow-up. Similar observations have been reported in larger studies evaluating telemedicine-based post-PCI care. In a multicenter Chinese randomized trial involving 2,086 patients following PCI, a comprehensive telemedicine platform incorporating health education, medication reminders, and artificial intelligence-assisted consultation significantly reduced the incidence of one-year major adverse cardiovascular and cerebrovascular events (MACCE) from 5.3% to 3.5% compared with telephone-based usual care [13]. This contrast highlights a central limitation of our trial and of most single-center digital-health studies in this space. With only 100 patients and single-digit event counts, our study was underpowered to detect the modest absolute risk reductions that considerably larger platforms have demonstrated for hard events [13,14]. Similarly, a randomized trial from the United Kingdom evaluating home-based smartphone-linked electrocardiographic and vital-sign telemonitoring after acute coronary syndrome required 337 participants to demonstrate a significant reduction in unplanned hospital readmissions, highlighting that adequately powered evaluations of telehealth interventions following PCI generally require substantially larger sample sizes than those included in the present study [14]. These findings are further supported by a systematic review and meta-analysis of telemedicine interventions across cardiovascular diseases, which demonstrated that reductions in mortality and hospitalization were most consistently observed among patients with heart failure receiving longer-term follow-up, whereas studies involving secondary prevention after coronary events more frequently reported improvements in intermediate cardiovascular risk factors rather than hard clinical outcomes over relatively short follow-up periods [15]. Likewise, a Cochrane review comparing home-based and center-based cardiac rehabilitation found no consistent differences in mortality or cardiovascular morbidity between the two delivery models across randomized trials [16]. Collectively, these findings are reassuring regarding short-term clinical outcomes with structured tele-follow-up following PCI, while emphasizing that the present study was not designed or sufficiently powered to establish non-inferiority with respect to major cardiovascular events.

One notable observation was the numerically higher incidence of heart failure-related hospitalization in the tele-follow-up group at one and three months, which accounted for most of the non-significant between-group difference in MACE during the early follow-up period. However, given the very small number of events involved (three to four hospitalizations among 50 patients), random variation remains the most plausible explanation. An alternative explanation, consistent with previous telemonitoring literature, is ascertainment bias. Patients receiving more frequent virtual contact with healthcare providers may have reported worsening congestive symptoms earlier, leading to hospitalization at a lower clinical threshold, whereas similar symptoms among patients receiving conventional follow-up may have remained unreported between scheduled outpatient visits. A comparable phenomenon has been described in studies of home blood-pressure telemonitoring, where closer surveillance resulted in more frequent medication intensification, reflecting more responsive clinical management rather than an increased incidence of adverse events [17]. Larger studies with longer follow-up and blinded adjudication of clinical events are required to determine whether this observation represents a true clinical signal or simply more complete symptom detection.

Medication adherence was high (96%-100%) and statistically similar between arms throughout follow-up. This near-ceiling adherence in both groups likely reflects the intensive, protocolized nature of post-PCI care common to both study arms, including structured counselling at enrolment and multiple scheduled contacts, rather than an absence of effect of the tele-follow-up modality itself. A meta-analysis evaluating mobile phone text-messaging interventions across chronic diseases demonstrated that such interventions significantly improve medication adherence when baseline adherence is suboptimal; however, the incremental benefit becomes considerably smaller once adherence approaches a ceiling, as observed in the closely supervised population included in the present trial [18]. Therefore, our findings are consistent with the existing evidence, with similarly high medication adherence observed in both structured tele-follow-up and conventional outpatient care, although the potential for further improvement was limited by the already high adherence rates.

Structured tele-follow-up also showed favorable numerical trends in lifestyle modification. Smoking prevalence declined more in the tele-follow-up arm by six months (6% vs. 18%), although the difference was not statistically significant, likely reflecting the modest sample size. Similarly, adherence to a healthy diet was consistently higher in the tele-follow-up group and approached statistical significance at three months (86% vs. 58%, p = 0.06). These findings are consistent with the TEXT ME trial, which demonstrated that lifestyle-focused text messaging improved smoking cessation and dietary behaviors among patients with CHD [19]. A systematic review of mobile health interventions similarly reported moderate improvements in lifestyle behaviors following coronary events [20]. Although statistical significance was not reached for all lifestyle outcomes, the observed trends suggest that regular virtual reinforcement may promote sustained behavioral change following PCI.

The most pronounced between-group difference in our study was observed in LDL-C control. Patients in the tele-follow-up arm had significantly lower mean LDL-C levels at one and six months, with a consistent, although non-significant, numerical advantage at three months and a higher proportion achieving the guideline-recommended LDL-C target of <55 mg/dL at every assessment. This finding is clinically relevant and aligns with a systematic review and meta-analysis demonstrating that mobile health interventions consistently improve lipid control following coronary events [20]. Its importance is further underscored by the EUROASPIRE V survey, which reported persistently poor LDL-C target attainment among more than 7,800 patients with established CAD despite widespread use of lipid-lowering therapy, highlighting a substantial evidence-practice gap in secondary prevention [21]. Although target attainment in the tele-follow-up group remained suboptimal at six months (56%), it exceeded that observed with standard follow-up, suggesting that structured tele-follow-up may help narrow this treatment gap. The observed benefit may reflect more frequent reinforcement of medication adherence, earlier identification of treatment discontinuation or adverse effects, and timely reminders for repeat lipid testing during virtual consultations. Similar mechanisms were demonstrated in the landmark MULTIFIT trial, where structured telephone-based case management produced significantly greater improvements in coronary risk-factor control, including LDL-C, than usual post-myocardial infarction care [22].

In contrast to the favorable effects observed for LDL-C, structured tele-follow-up did not confer a measurable advantage in glycemic or blood pressure control. Mean HbA1c values remained comparable between groups throughout follow-up, suggesting that glycemic optimization, unlike lipid control, may require active medication titration rather than behavioral reinforcement alone. Similarly, blood pressure outcomes showed no sustained between-group differences despite a transient reduction in diastolic blood pressure at one month in the tele-follow-up arm. These findings differ from those of home blood pressure telemonitoring studies, in which device-enabled monitoring combined with protocol-driven medication adjustment significantly improved blood pressure control [17]. The absence of integrated home-monitoring devices and structured treatment-titration algorithms in our intervention likely explains these findings and is consistent with the SMARThealth India trial, which likewise demonstrated no significant improvement in blood pressure target attainment despite mobile health-supported care [23]. Together, these observations suggest that tele-follow-up alone may be sufficient to reinforce behaviors that depend largely on patient adherence, but may be inadequate for outcomes requiring active therapeutic intensification [24].

This interpretation is further supported by the overall pattern of results observed in the present study. Behavior-dependent outcomes, including LDL-C control, dietary adherence, smoking cessation, and maintenance of medication adherence, consistently favored structured tele-follow-up, whereas hard clinical outcomes, glycemic control, and blood pressure remained comparable between groups. Baseline demographic, clinical, and biochemical characteristics were generally comparable between groups, although current alcohol consumption was higher in the standard follow-up arm. This difference most likely reflects a chance imbalance related to the modest sample size, a recognized feature of smaller randomized trials [25]. Although not statistically significant, patients assigned to tele-follow-up also resided farther from the study hospital, reinforcing the practical value of telehealth in settings such as India, where travel distance and limited access to specialist cardiac services remain important barriers to long-term secondary prevention [23]. Collectively, these findings suggest that structured tele-follow-up should be viewed as a complementary strategy to conventional post-PCI care, with its greatest value lying in reinforcing adherence and promoting sustained lifestyle modification. Future programs incorporating remote physiological monitoring, protocol-driven medication titration, and longer follow-up may achieve broader improvements in cardiovascular risk-factor control and clinical outcomes.

Limitations

This study has several limitations. It was a single-center, open-label study with a relatively small sample size and a follow-up period of six months. The open-label nature of the study may have introduced some degree of performance and ascertainment bias, particularly for subjective outcomes. In addition, information on medication adherence and lifestyle practices was largely based on patient reporting and may therefore be affected by recall or reporting bias. Clinical events were not independently adjudicated by a blinded endpoint committee. Some participants were lost to follow-up, and the analysis was based on available data without imputation for missing outcomes. Several secondary outcomes were assessed at multiple time points without adjustment for multiple comparisons; therefore, isolated statistically significant findings should be interpreted with caution. The small number of clinical events also limited our ability to detect meaningful differences in MACE and other infrequent outcomes. Furthermore, tele-follow-up required access to a smartphone and internet connectivity, which may limit its applicability in populations with poor digital access or literacy. Finally, higher-risk patients, including those with LVEF <40%, symptomatic heart failure, or severe anemia, were excluded. Our findings therefore primarily apply to a selected, relatively stable post-PCI population and may not be generalizable to higher-risk patients. Larger multicenter studies with longer follow-up and more diverse patient populations are needed to confirm these findings.

## Conclusions

In this small, randomized, single-center study, structured tele-follow-up was associated with comparable observed short-term clinical outcomes and lower mean LDL-C levels compared with standard follow-up. Although no statistically significant between-group difference in MACE was observed during the six-month follow-up, these findings should not be interpreted as evidence of equivalence or non-inferiority. The observed reduction in mean LDL-C at selected follow-up time points represents a potential benefit of structured tele-follow-up, while differences in other cardiovascular risk-factor outcomes were not consistently statistically significant. These findings support the feasibility of structured tele-follow-up as a follow-up strategy after PCI, but larger, multicenter studies involving broader and higher-risk populations with longer follow-up are warranted to confirm its clinical effectiveness and generalizability.

## References

[1] Lindstrom M, DeCleene N, Dorsey H (2022). Global burden of cardiovascular diseases and risks collaboration, 1990-2021. J Am Coll Cardiol.

[2] Chong B, Jayabaskaran J, Jauhari SM (2025). Global burden of cardiovascular diseases: projections from 2025 to 2050. Eur J Prev Cardiol.

[3] Kalra A, Jose AP, Prabhakaran P (2023). The burgeoning cardiovascular disease epidemic in Indians - perspectives on contextual factors and potential solutions. Lancet Reg Health Southeast Asia.

[4] Prabhakaran D, Jeemon P, Roy A (2016). Cardiovascular diseases in India: current epidemiology and future directions. Circulation.

[5] Prabhakaran D, Jeemon P, Sharma M (2018). The changing patterns of cardiovascular diseases and their risk factors in the states of India: the Global Burden of Disease Study 1990-2016. Lancet Glob Health.

[6] Xavier D, Pais P, Devereaux P (2008). Treatment and outcomes of acute coronary syndromes in India (CREATE): a prospective analysis of registry data. Lancet.

[7] Sharma M, Ganguly NK (2005). Premature coronary artery disease in Indians and its associated risk factors. Vasc Health Risk Manag.

[8] Gupta R, Mohan I, Narula J (2016). Trends in coronary heart disease epidemiology in India. Ann Glob Health.

[9] Ryu S (2012). Telemedicine: opportunities and developments in member states: report on the second global survey on eHealth 2009 (global observatory for eHealth series, volume 2). Healthc Inform Res.

[10] Ekeland AG, Bowes A, Flottorp S (2010). Effectiveness of telemedicine: a systematic review of reviews. Int J Med Inform.

[11] Fatehi F, Gray LC, Russell AW (2013). Telemedicine for clinical management of diabetes - a process analysis of video consultations. J Telemed Telecare.

[12] Scott Kruse C, Karem P, Shifflett K, Vegi L, Ravi K, Brooks M (2018). Evaluating barriers to adopting telemedicine worldwide: a systematic review. J Telemed Telecare.

[13] Yu X, Cao J, Xu J (2025). Efficacy of telemedical interventional management in patients with coronary heart disease undergoing percutaneous coronary intervention: randomized controlled trial. J Med Internet Res.

[14] Alshahrani NS, Hartley A, Howard J (2024). Randomized trial of remote assessment of patients after an acute coronary syndrome. J Am Coll Cardiol.

[15] Kuan PX, Chan WK, Fern Ying DK (2022). Efficacy of telemedicine for the management of cardiovascular disease: a systematic review and meta-analysis. Lancet Digit Health.

[16] Taylor RS, Dalal H, Jolly K, Moxham T, Zawada A (2010). Home-based versus centre-based cardiac rehabilitation. Cochrane Database Syst Rev.

[17] Omboni S, Guarda A (2011). Impact of home blood pressure telemonitoring and blood pressure control: a meta-analysis of randomized controlled studies. Am J Hypertens.

[18] Thakkar J, Kurup R, Laba TL (2016). Mobile telephone text messaging for medication adherence in chronic disease: a meta-analysis. JAMA Intern Med.

[19] Chow CK, Redfern J, Hillis GS (2015). Effect of lifestyle-focused text messaging on risk factor modification in patients with coronary heart disease: a randomized clinical trial. JAMA.

[20] Cruz-Cobo C, Bernal-Jiménez MÁ, Vázquez-García R, Santi-Cano MJ (2022). Effectiveness of mHealth interventions in the control of lifestyle and cardiovascular risk factors in patients after a coronary event: systematic review and meta-analysis. JMIR Mhealth Uhealth.

[21] De Backer G, Jankowski P, Kotseva K (2019). Management of dyslipidaemia in patients with coronary heart disease: results from the ESC-EORP EUROASPIRE V survey in 27 countries. Atherosclerosis.

[22] DeBusk RF, Miller NH, Superko HR (1994). A case-management system for coronary risk factor modification after acute myocardial infarction. Ann Intern Med.

[23] Peiris D, Praveen D, Mogulluru K (2019). SMARThealth India: a stepped-wedge, cluster randomised controlled trial of a community health worker managed mobile health intervention for people assessed at high cardiovascular disease risk in rural India. PLoS One.

[24] Jin K, Khonsari S, Gallagher R (2019). Telehealth interventions for the secondary prevention of coronary heart disease: a systematic review and meta-analysis. Eur J Cardiovasc Nurs.

[25] Roberts C, Torgerson DJ (1999). Understanding controlled trials: baseline imbalance in randomised controlled trials. BMJ.

